# Supplementary material for: KMT5C leverages disorder to optimize cooperation with HP1 for heterochromatin retention
Source: EMBO Rep. 2024 Nov 19;26(1):153–74. doi: 10.1038/s44319-024-00320-5 (PMC11723951; doi:10.1038/s44319-024-00320-5)
Supplement: Supplementary file 4 — Dataset EV3 [file 44319_2024_320_MOESM4_ESM.docx]

Dataset EV3

**FASTA files for representative mammalian, bird, reptile, and frog KMT5C orthologs. Hydrophobic motifs are underlined and the region spanning the histidine-cysteine motifs is in bold.**

>Homo_sapiens

MGPDRVTARELCENDDLATSLVLDPYLGFRTHKMNVSPVPPLRRQQHLRSALETFLRQRDLEAAYRALTLGGWTARYFQSRGPRQEAALKTHVYRYLRAFLPESGFTILPCTRYSMETNGAKIVSTRAWKKNEKLELLVGCIAELREADEGLLRAGENDFSIMYSTRKRSAQLWLGPAAFINHDCKPNCKFVPADGNAACVKVLRDIEPGD*EVTCFYGEGFFGEKNEHCECHTCERKGEGAFRTRPREP*ALPPRPLDKYQLRETKRRLQQGLDSGSRQGLLGPRACVHPSPLRRDPFCAACQPLRLPACSARPDTSPLWLQWLPQPQPRVRPRKRRRPRPRRAPVLSTHHAARVSL**HRWGGCGPHC**RLRGEALVALGQPPHARWAPQQDWHWARRYGLPYVVRVDLRRLAPAPPATPAPAGTPGPILIPKQALAFAPFSPPKRLRLVVSHGSIDLDVGGEEL

>Mus_musculus

MGPDRVTARELCENDDLATSLVLDPYLGFRTHKMNVSPVPTLRRQHHLRSALEAFLRQRDLEAAFRALTLGGWMAHYFQSRAPRQEAALKTHIFCYLRAFLPESGFTILPCTRYSMETNGAKIVSTRAWKKNEKLELLVGCIAELREEDEDLLRAGENDFSIMYSTRKRSAQLWLGPAAFINHDCKPNCKFVPSDGNTACVKVLRDIEPGDEVTCFYGEGFFGEKNEHCECYTCERKGEGAFRLQPREPELRPKPLDKYELRETKRRLQQGLVSSQQSLMSRWACSHLSPLRPDPFCAACQPSCLLPASPHMDYLPLWLQRAPQPQPIVPPRKRHRRRRPRIRQASLPPVLRTACVPL**HRWGGCGPHC**QLRAEAMVTLHLRPQTRWTPQQDWYWARRYGLPSVGRVELTRLAPALPAAPAPAGNPGPVPTPDFIPKQALAFAPFCPPKRLRLVVSHGSIDLDINSGEP

>Canis_familiaris

MPSRPSVVRMRLETFKSLAKLRAAPKWHSWGTAPAPTLCPPGQRQHGPWGTMGPDRVTARELCENDDLATSLVLDPYLGFRTHKMNVSPVPPLRRQHHLRSALEAFLRQRDLEAAYRALTLGGWMAHYFQSRGPRQEAALKTHIYRYLRAFLPESGFTILPCTRYSMETNGAKIVSTRAWKKNEKLELLVGCIAELREADEGLLRAGENDFSIMYSTRKRIAQLWLGPAAFINHDCKPNCKFVPADGNAACVKVLRDIEPGDEVTCFYGEGFFGEKNEHCECYTCERKGEGAFRLRPREPLPPRPLDKYELRETKRRLQQGPDRSRRQGPLGPRACAHLSPLHRDPFCAACQPLRPLPCGTRPDASPLWLQWLSQPQLRARPRRRRRPQPRRAPALPVLRAARVSL**HQWGGCGPRC**CLRAEALVALGPAPRAHWAPQQDWHWARRYGLPYVVRVDLSRVAAALPATPVPAPVGTPGPTPFPKQALAFAPFSPPKRLRLVVSHGSIDLDVNGDGP

>Bubalus_bubalis

MGPDRVTARELCENDDLATSLVLDPYLGFRTHKMNVSPVPPLRRQHHLRSALEAFLRQRDLEAAYRALTLGGWMAHYFQSRGPRQEAALKTHIYRYLRAFLPESGFTILPCTRYSMETNGAKIVSTRAWKKNEKLELLVGCIAELREADEELLRAGENDFSIMYSTRKRSAQLWLGPAAFINHDCKPNCKFVPADGNAACVKVLRDIEPGDEVTCFYGEGFFGEKNEHCECYTCERRGEGAFRLRPREPLPPRPLDKYELRETKRRLQQGLDGPRSCAHPPPLRRDPFCAACQPLRPPPCSARLDASPLWLHWLPQPQLRVRPRRRRRLRLQSRRALPPPVLRAARVSL**HRWGGCGPHC**RLRAEARVALVQAPRARWAPQQDWHWARRYGLPSVVRVNLSRVVPALPATANPAPSGTPGPVPVPKQALAFTPFSPPKRLRLVVSHGSIDLDVNSDGP

>Cavia_porcellus

MPSCAPRLPAVYGRQHGSQNSMGPDRVTARELCENDDLATSLVLDPYLGFRTHKMNVSPVPTLRRQQHLRSALEAFLRQRDLEAAYRALTLGGWMDHYFQSRGSRQEAALKTHVFRYLRAFLPESGFTILPCTRYSMETNGAKIVSTRAWKKNEKLELLVGCIAELREADEDLLRAGENDFSIMYSTRKRSMQLWLGPAAFINHDCKPNCKFVPADGNAACVKALRDIEPGDEVTCYYGDGFFGEKNEHCECYTCERKGEGAFSLKPRDPEMPPSQTPDKYAFRETKCRLQRGQDSGRQSLLGPRACTHLFPLRWDPFCAVCQPLRLLPSISPLDSLPLWLQWLPQPQPRARPRKRRRPRPQQAFALPVLREAQVSL**HPWGGCGPHC**RLRGEALVALGLPHSARWAPQQDWHWARHYGLPSVVRVDLSRLDPLPAPATAPALVTPAASAHSGNPGSIPASVPVPKQALAFASFSPRRRLRLVVSHGSIDLDVNSDEL

>Coturnix_japonica

MRRDGGRTRVAARAAGGPGEVRPRPARYRDQDPTLDPGSLQKDAPDGAAMGSRRLTAKELCEIDDLATSLVLDSVLGFRTHKMGVSPLPALRRKHLLRAAVEGFRRRRDLEAAWRALSGAWAAAFFQRRRPLQRAALKSHVFHYLRIFLPESGFSIQPCSRYSQETNGARVVSTRTWYLLRQKNDTLELLVGCIAELRAHDEELLRSGENDFSIMYSTRKRCAQLWLGPAAFINHDCRPNCKFVPAEGNVARVQVLRDIAPQDEITCFYGDSFFGDNNERCECCTCERKGEGAFRKLGRGAQPLPPPTPPKYLLRETDRRLRRARGLGAARARPHRQRRRRLRGTRHPPAPRPPQLRVAI**HDCISCGGGC**LLQGKEPLVCVCRLQPPEPPPPQPTTTATPAVLPPREDPKLSLYAHVRLGPLGGSCGGGTALMSPQKHPGAFGATGRLRLVVTHGSIALDVAPNVPPSV

>Phasianus_colchicus

MMREGGRRRVAARAAAEQREEQGRPGPDPRSSRAPPAAPPAALRAQDRTLDPDFSVKDAPRGTAMGSRRLTARELCEIDDLATSLVLDSVLGFRTHKMGVSPLPALRRKHLLRAAVEGFRRRRDLEAAWRALSGSGAWAAAFFQRRRPLQRAALKSHVFHYLRIFLPESGFAIQPCSRYSQETNGARVVSTRTWQKNDTLELLVGCIAELRAHDEALLRSGENDFSIMYSTRKQCAQLWLGPAAFINHDLLADRCCCFPLFQTAGPTASLYLQKGTWHGCRCCGTSRRRMRSPASMGTASLGTTTSAASAAPARASPCRKGEGAFRKRKGGAQPVPPLTPPKYLLRETDRRLCRARASGGTRSHPHRQHRRRRLRGTSRPPAPRPPQLRVVL**HDCISCGGGC**RLQGREPLVCLCRLPPPEPPAAAAAPRPTPPTPSLLFPLQDPKLSLYAHVRLGPAGGSCGGGTAMMSPQKHPGAFGATGRLRLVVTHGSIALDVAPDVPSV

>Gallus_gallus

MGSRRLTARELCEIDDLATSLVLDSVLGFRTHKMGVSPLPALRRKHLLRAAVEGFRRRRDLEAAWRALSGAWAAAFFQRRRPLQRAALKSHVFHYLRIFLPESGFAIQPCSRYSQETNGARVVSTRTWQKNDTLELLVGCIAELRAHDEALLRSGENDFSIMYSTRKQCAQLWLGPAAFINHDCRPNCKFVPAEGNVARVQVLRDIAPQDEITCFYGDSFFGDNNERCECCTCERKGEGAFRKRSGGAQPVPPLTPPKYLLRETDRRLRRARGPGGARGHPHRQHRRRRRLRGASRPPAPRPPQLRVVL**HDCISCGGGC**RLQGREPLVCLCAAAPAGAPPPQPTAATPPAVLPPLEDPKLSLYAHVRLGPAWGSCGGAQL

>Numida_meleagris

MGSRRLTARELCEIDDLATSLVLDSVLGFRTHKMGVSPLPALRRKHLLRAAVEGFRRRRDLEAAWRALSGAWAAAFFQRRRPLQRAALKSHVFHYLRIFLPESGFAIQPCSRYSLETNGARVVSTRTWQKNDTLELLVGCIAELRAHDEALLRSGENDFSIMYSTRKQCAQLWLGPAAFINHDCRPNCKFVPAEGNVARVQVLRDIAPQDEITCFYGDSFFGDNNERCECCTCERKGEGAFRKRSGGAQAVPPPSPPKYLLRETDRRLRCAKGPGGARGHPHRQHRRRRRRRLRGTSRPPAPRPPQLRVVL**HDCISCGEGC**RMQGREPLVCLCRQPPAAPLPEPPPPQPAAAAPTTTTVLPTLEDPKLSLYAHVRLGPAGGSSRGGPAMISPQKHPGAFGATGRLRLVVTHGAIALDVAPGVLPSI

>Catharus_ustulatus

MRSRTPVAAVTARELCENDDLATSLVLDSVLGFRTHKMGVSPLPALRRRAQLRAAIEAFRQRRDLEAAWRTLSAAWAPAFFRQRRPVQRAALKIHVFRYLRTLLPESGFSIQRCTRYSHDTNGARVVSTRTWRKHETLELLGGCAVELRGSDRALLRAGDNDFSLMYSTRRRRTQLWLGPAAFINHDCRPNCRFVSSPGGARVQALQDIPPRAEITCFYGDGFFGEGNLGCECRTCERRGEGAFRHRGKMAEPPPQDPPPKYELRQTDVRLRRGGAGQDGTPPHNPPRRKRPPPANRKRRHLGRHLVPPSRRGRHLGRGGAEPRVSL**HDCVSCGSRC**RLRGGVPVVRLGGLETPKPLPKSVPAPRPDPKLSLYAHVRLGGGAPKLEEGEDRGSPPRLRLVVTQGGLALTLPPLSEH

>Anolis_carolinensis

MGSTRVTAKELCEIDDLATTLILDPYVGFKTHKMNVSPLPAIRRKHHLREAVEAFRKRKDLEAAYQTLMQGGWACQYFANRSRQQEAAFKTHIFRYLRIFLPESGFVIKRCNRYSLEINGARVVSAKSWRKNDKLELLEGYIADLTEQDESLLRTGENDFSVMYSTRKQSAQLWLGPASFINHDCRPNCKFVPMDGNIACVKVLRDIEPDDEITCFYGDGFFGENNELCECYTCERKGGGAFRLLNQTPSPSISDNEKYLLRETDGRLQRWKNQSCKLAQRSGKVGRKTRRRRARLRGTLRRSSIRWYFRMLKPLYIPV**HDCLACSAHRC**KIHKQLLVRLIRCPPSMLPVSHLRHSRRSRVQQDYSFMKKHNMTWLDRRSLVVNLGKRVPLDWVGLQTDKVSENGDHQLHSSNVELSQGIEEHPTGTVENHTEVTEPLASGEQAPFRSSDPLSSSQLLYRTRSMARRQAKLLPKSQLALPAVDPKLAQYAHVNLEVPPLEGSEHWEQPPPDPLEKEQVEEQVEEQVEEQPPPKQMVSFPPFVPPKRLRLVVSHGSINLEMASGSCEELS

>Pogona_vitticeps

MGSTRVTAKELCENDDLATTLILDSYLGFKTHKMNVSPLPAIRRKHHLREAVEAFRKRKDLEVAYQALMQGGWACQYFANRSRQQEAAFKTHIFRYLRIFLPESGFVIKRCSRYSLEINGARVVSTKSWRKNDKLELLEGYIAELTEEDESLLRTGENDFSIMYSTRKQCAQLWLGPAAFINHDCRPNCKFVPMDGNIACVKVLRDIEPEDEITCFYGDGFFGENNELCECYTCERKGEGFFRLRNQTPSQSTSVNEKYLLRETDGRLQRWKSQSCKLAQRSVKVGQKARRRRARLRGALRRSTLRYYFRMLKPLYIPL**HDCLACGARQC**KIHKQLLVRLLQCPPSMLPASHLRRSLRSKARYSRAPQGCSVTEKHKSPSSSWTNQCNLVVNLGKRVPLNLWMNRKAVSGTGQREPYSRNGAFSQGPKERHEGDEEVTGPYESGEQAPLGSSGPLSYSQLLCRTRSMAKRQAQLLPKSQLFVPAVDPKLSQYAHVCLEGPLFGGRQRWQQPPPEEVEKEQAEEQQPPSKPMVSFPPFVPPKRLRLVVSHGSINLEVASGSCEELS

>Lacerta_agilis

MGSTRVTAKELCENDDLATTLILDSYLGFKTHKMNVSPLPAIRRRHHLREAVEAFRKRKDLDMAYQALMQGGWACQYFAKRSRQQEAAFKTHIFRYLRIFLPESGFAIKRCTRYSLEINGARVVATKSWKKNDKLELLEGYITELTEPDESLLRTGENDFSIMYSTRKQCAQLWLGPAAFINHDCRPNCKFVPMDGNTACVKVLRDIEPDDEITCFYGDGFFGEDNELCECYTCERKGEGAYRLLNQTPPQSTSINDTYLFRETDGRLQRWKRPSYKLARLSVKVGSNARRRKAKIRAALRRSALRWYLRILKPLCIPL**HNCLGCTACSC**KLSKQPLVHLQRCPPSMFPASHLRRSSRKKSSHFETQQSFFITRTYNRPESPSWADQSDLVVNLGKRVPLHWEGLHRQAVRGIGEREPQSSNGELSQGAEEHHTGDVKNPAEIMGSHGSGSGELYSGDQEILGSTSSSSSGQVLCRTRSMTRRQTWVLPKPQVSLSAVDPKLSQYAHVRLESPLFRGRQCWEQPPPDKVKEELTEEQHPPKQIVSFPPFVPPKRFRLVVSHGSINLEVASSSCEELS

>Podarcis_muralis

MGSTRVTAKELCEIDDLATTLILDSYLGFKTHKMNVSPLPAIRRKHHLREAVEAFRKRKDLDMAYQALMQGGWACQYFAKRSCQQEAAFKTHIFRYLRIFLPESGFAIKRCTRYSLEINGARVVATKSWKKNDKLELLEGYITELTEPDESLLRTGENDFSIMYSTRKQCAQLWLGPAAFINHDCRPNCKFVPMDGNTACVKVLRDIEPDDEITCFYGDGFFGEDNELCECHTCERKGEGAYRLLNQTPPQSTSINDNYLLRETDGRLQRWKRPSYKLARLSVKVGCKARRRKAKIHAALRRSAFRWYLRILKPLCIPL**HNCLGCTARSC**KLSKQPLVHLQRCPPSMFPASHLRRSSRRKSSHFETQQSFFFTRTYNRPESPSWADQSDLVVNLGKRVPLHWEGLRREAVRGIGEREPHSSNGELSQGAEEHHTGDVKNPAEIMGSHGSGSGELYSGDQEILGSTGSSSSGQVLCRTRSMTRRQTWDLPKPQVSLLAVDPKLSQYAHVRLESLLFRGRQCWQQPPPDKGKEEPTEEHPPPKQIVSFPPFVPPKRFRLVVSHGSINLEVASSSCEELS

>Python_bivittatus

MGSTRVTAKELCENDDLATTLILDSYLGFKTHKMNVSPLPAIRRKHHLREAVEAFRKRKDLEAAYQALMQGGWACQYFANRSHQQEAAFKTHIFRYLRIFLPESGFAIRRCSRYSLEINGARVVSTKSWRKNDKLELLEGYIAELTEPDESFLRAGENDFSIMYSTRKQCAQLWLGPAAFINHDCRPNCKFVPMDRNTACVKVLRDIEPDDEITCFYGDGFFGENNELCECYTCERKGEGAFRLLNQTPLLSTSVNEKYLLRETDGRLQRWKNQSRKLAQRSVKVGHKARRRKSQLHGAVHRYTTRWYPRMLKPLYIPL**HNCLACRARRC**KLRKQPLVCLLRCPPPVLPVLHLRQSLRNKNCYSKAQQHCSLIKVHSCPGSLGLAGQCSLVVNLGKRIPLDWARFHREVVCDVKERKPPITNGEFSQGTEEYDSRDMESHADVIETRESGSGVLHSGQQVPLDSSGPLPSRQFLCRTRSMAQRQAQVLPRSQLSLATVDPKLSQYAHVRLEGPLFGGRQCWQHSSSDKMEKEQAEEQHLPKRMVSFPPFVPPKRLRLVVSHGSINLEVAASSCEELS

>Alligator_sinensis

MGSTRVTAKELCENDDLATSLVLDSYLGFKTHKMNVSPLPAIRRQHHLREAVEAFRRRRDLEAAYRALMLGDWSCQYFQNRSCQQEAALKIHIFRYLRIFLPESGFTILPCSRYSLETNGARVVSTKSWRKNDKLELLVGCIAELTEPDESLLRAGENDFSIMYSTRKRCAQLWLGPAAFINHDCRPNCKFVPTEGNTACVKVLRDIEPQDEITCFYGDGFFGDNNELCECCTCERKGEGAFRQQQKDPSQATSAQEKYQLRETDGRLQRWKGRVSKKVQPGAKAARRARRRRDRLRALRACQLQPGWTPSLPCFPQLQALRHLRIPL**HNCVACRGIGRGC**RLRKELAVSLVRCLPGASAPETAVAQSSEALEHHPALYQHPPSSTEPSIPSSLGSLDLLRLKTPSLTRVFCVELGDTRVLASQDRGQGPGIGPPMLGVSSTPGEASPGAVPHSRDVVPPGGVVGHCPLAPQLLCRTRSMVKAVASCPAWPLEALAAAGRANPHPLPDPKLSLYAHVRLEKTEQPPQPAMSRTEPEEASAPSGDPKRTVTFQPFPPAKRLRLVVSHGSIDLDLASAASEEAA

>Xenopus_laevis

MGSNRLTARELCENDDLATSLVLDPYLGFRTHKMNVSAMPTIRRQHHLREALQTFHKKKDPEAAYQSLTAGEWARHYFHSRSRQQESLLKAHIFRYLRMFLPESGFMILSCSRYSLETNGAKVVSTKSWSKNEKIELLVGCIAELSKADETLLRFGDNDFSVMYSTRKKCAQLWLGPAAFINHDCRPNCKFVPTDGNAACVKVLREIKSGEEITCFYGDSFFGEKNEQCECCTCERKGEGAFKQQKTEQTVSTSLEKYQLRETDGRLKRLRESACKQSHQVTTKKKKRPQGSKVRLALRLRRIPPSRRKRVFYRRLRTLASSHFYSSSLAKHISFKPVKIALPRGTVLRDVRIIL**HNCKRCNMASRSKSQHEGKCC**KLGKVPLVSILREDLSPEKLKFRLICPPTIQTANIECNAKDRSLPEAGSINMEPITTAEVCEHVSFSPLPSHNGDEDSFYEPEIPGSLLGPESPSSVLESGSEDGLSGPNNDEPISNNLNMECPPLEPIVKDTVYPYDGSKMSVSHLHALKQFGITRYIKVDLRKHVDLQSEKSQPDKSSSAANRKLNPNHIHSHQPLRSDDHLLANLNAKAQNKVVLRKDVALESEKPRPDKSSSATNRKPNLNHIHSQLPIRSDDHLPANFNTEAQNNTVVSPTAKTPISEALNGSPDRKVSSLRSRSVTFRETSDNKITLFKRRRSSVKPGVQCVKLNGHVKLTGQIVYSKLDPPTKAVAKHLTHTMHSDPKLLLKPYVQLGINNNLKRSSFTGLAHSKVLTEEAVKLHNPPASKPSTVGAKKNVAFNPFTPSKRLRLVVSLGSIALDMASTSSEETN
> Xenopus_tropicalis

MGSNRLTARELCENDDLATSLVLDPYLGFRTHKMNVSAMPTIRRQHHLREALQTFRKKKDLEAAYHSLAAGEWARHYFQSRTRQQESLLKTHIFRYLRMFLPESGFMILSCCRYSLETNGAKVVSTKAWCKNEKIELLVGCIAELSKADETLLRFGDNDFSVMYSTRKKCAQLWLGPAAFINHDCRPNCKFVPTEGNTACVKVLREIKSGEEITCFYGDSFFGEKNELCECCTCERKGEGAFKQQKTEQTVSTSLEKYQLRETDGRLNRLSESACKQSQQVTARRKKDPPGSRVRLSLRLKRIPASRRKRAFYRRLRTLASSRYFYSSRLVKHIPSKPVKIALPQGTVLRDVRIIL**HNCKKCNMASHPKHQHDGQCC**KLGKEPLVSLRREDLSPEKLKFRLSCHGGSCSPTIQTANIECNAKDCPLPEAEKASAEQVTSAEVCEHLSFSPVPSLSGDDHSFYEPEIPASLLGPDSPSAEPLVKEPLSSVCPPPTVYPHNNNNGDIMSALHHQALKQFGITHYIKVGLSKDVRSLEKSGKNPADSSSLAAEPSDNCIHSQHPVTSDDRLAGNLTDETQGNPVSSPAKTQRCEALNGSLAPKLFSLRSRPVSFKASETSDNKITLFKRSSSSSAKPGVQYVKLNGHVKLTGQLVPSEPHAPNAARARPLADTMRSDPKLLLKPYVELGLNNNLKRHSVTGLPPSAVLTEEAFNKLHSSPASKQLAEGAKKKVAFNPFTPSKRLRLVVSHGSIALDIASTSSEETA

>Hymenochirus_boettgeri

MGSNRITAKELCENDDLATSLVLDPYLGFRTHKMKVSAMPAIRRQHHLREALQTFLKKKDLEAAYRSLTTGEWARQYFQNRTRQQESLLKAHIFRYLRMFLPESGFMILSCGRYSLESNGAKVVSTKSWVKNEKIELLVGCIAELSKADESLLRFGENDFSVMYSTRKKCAQLWLGPAAFINHDCRPNCKFVPTEGNAACVKVLREIRPDEEITCFYGDSFFGEKNELCECCTCERKGEGAFKLQKAEQTVSTSLEKYQLRETDGRLKRLSNSICKQIHQEATKRHKPHQGSKYRLSVNFKRIPATCKRQAFSQRLRTPASSPYYYYCSNIVKHIPSKRMISLKPVRVALPRGTVLRDIRVIL**HNCKKCHATSHFQHLHERQCC**KLGKEPVVSLLREDLSPEKLKFRLHCSATYEASLTTPTVRFDRNAKDVCLTEVEVGESMVEQDTIAKVCESLSFSSVLSHSSDDDTFSEPEMPGSFLGPGSPSSDPPVQIQEAESNTIDTDFFPFESAAKDTVHPQNNADVSQFTLDSLNKQFGITHYVTVDLGKSIKRGVFNNSLAANVKPRDDHGLLPPAENTNGNLLASLDNETQSHTVNEPNKSKKNEALSESLSHKALSLRSRPVILKEQEASENSISLKQKRSCVNPNLKCLKINGHVKVTGQVVNSEPHQVKVAKTKNLNDPLNLDPKLLLKPYVELGMNNNLKRCSAKGELCPRVVAKNSFGKLGQPQASTHLVDNAKQNVAFSSFTPSKRLRLVVTHGSIALDIASTSSEETS

>Rana_temporaria

MGSNRVTARELCENDDLATSLVLDPLVGFRTHKMNVGPLPSIRRQHHLREALHTFRKKRDLEAAFRALITGANHYLKNQITQQEATLKAHIFRYLRMFLPESGFMIRSCNRYSMEANGAKVVATKPWVKNEKIALLVGCIAELTKADESLLRFGENDFSVMYSTRKKCAQLWLGPAAFINHDCRPNCKFVPSDGNAACVKVLRDIKPDDEITCFYGDSFFGEKNECCECCTCERKGEGAFRLLKKDPSESTSSEKYQLRETDGRLQRLQGQSDKQTQHGNKRKRVSGSRCRSSPSLKKSPVNSKNSIFLSRLRSPFSSPYSRSCHRAQRSPINRSQMPVKFVLPPGTIIRDIRINL**HNPKCSRYSSAAGTPSEVHGC**KLSKEPVVRLSRQNVSPDKLSFYQAVDRALHNSSRTSLQGKTNALEKNAKDHTLNCALFNEPNVVEEHLDLEYLSATTSCCRDELDVDVYENQNCVSDSISNSDRLSTNGTDISMVQPEFDILASPSNSNRLCSAVVSQMQDDNFPPAASLPENTVNLNNSSFKLFSDSVSLKQLGLTHYVTVNLSKSRVSGAEYSNTPSFFHSDVRKHKRNRNQKPFTGFSHDQIVTKNANESPVIAENGSLTEPADINVKPRTGTCTNEVFSLQSTPTMFKVPNKRRKTTFHKKRRTTHLDFNGLIKVTGKHRISRPERRHSAPHALLSIEMQSDPKLSLKPYVELGLNNNLKRKVSVSEAQTCPVPLTEGAADKSMFISSQSELLANGNKRKNVTFSPFTPSKRLRLVVNNGSIDLDIASSASDESN

>Nanorana_parkeri

MGSTRLTARELCENDDLATSLVLDPYLGFRTHKMNVGPLPSIRRRHHLREALHTFRRKRDLEAAFRAFITGANHYLKNQIPQQEATLKAHIFRYLRMFLPESGFTILPCNRYSLETNGAKVVATKPWAKNDKIALLVGCIAELTKADESLLHFGENDFSVMYSTRKRCAQLWLGPAAFINHDCRPNCKFVPTEGNTACVKVLRDIKPEEEITCFYGDSFFGEKNELCECCTCERKGEGAFLLQKKGPCESTSSEKYRLRETDGRLRRLQGKSDRQTQRGTTRKRKRVLSLRCRSSPSLKKSPGNCKNSTFQPRLRSSASSQCFHSCHGAQCSLLYRQQRPVKFALPPGTIIRDVRINL**HNSIKCSRYSSAAATPCEVHGC**KLGKEPVVRLRRQNASPDRLRFYQGSDNVLHKSSQTSLHGKTDTLEKDAKDHTLNCVLFNEHSVMEECLNPECPSASTSVCVDELDIEVIEHHENCASNFISNSDRLNANGAHTSMVQPALDVLVSPLDSNRLCSNVVSQMQEADNFPPARSLPESTVNLNENSSRLTFSDSMSPKQFGLTHYVTVNLSKFRVSGPEYSSTASAFHSDIGKHKRSYNQKPFTGITHEAVLATNAIESQVMDANSSLTEPVGINVKHSTRTYTNKVFSLQSRPIMSKGLNEKSASHKKRQTTHLDFNGHVKVTGKHQSSRLERHHSAPHKLLSMEMQSDPKLSLKPYVELSLNNNLKRKVSVSEAQMCPVPFTEGASNKSVFISPQSELLTNSSKKKNVTFSPFTPSKRLRLVVTNGSIDLDIASSASDESN

**FASTA files for HRD sequences**

>Homo_sapiens

THHAARVSL**HRWGGCGPHC**RLRGEALVALGQPPHARWAPQQDWHWARRYGLPYVVRVDLR

>Mus_musculus

VLRTACVPL**HRWGGCGPHC**QLRAEAMVTLHLRPQTRWTPQQDWYWARRYGLPSVGRVELT

>Canis_familiaris

VLRAARVSL**HQWGGCGPRC**CLRAEALVALGPAPRAHWAPQQDWHWARRYGLPYVVRVDLS

>Bubalus_bubalis

VLRAARVSL**HRWGGCGPHC**RLRAEARVALVQAPRARWAPQQDWHWARRYGLPSVVRVNLS

>Cavia_porcellus

VLREAQVSL**HPWGGCGPHC**RLRGEALVALGLPHSARWAPQQDWHWARHYGLPSVVRVDLS

>Coturnix_japonica

RPPQLRVAI**HDCISCGGGC**LLQGKEPLVCVCRLQPPEPPPPQPTTTATPAVLPPREDPKLSLYAHVRLG

>Phasianus_colchicus

RPPQLRVVL**HDCISCGGGC**RLQGREPLVCLCRLPPPEPPAAAAAPRPTPPTPSLLFPLQDPKLSLYAHVRLG

>Gallus_gallus

RPPQLRVVL**HDCISCGGGC**RLQGREPLVCLCAAAPAGAPPPQPTAATPPAVLPPLEDPKLSLYAHVRLG

>Numida_meleagris

RPPQLRVVL**HDCISCGEGC**RMQGREPLVCLCRQPPAAPLPEPPPPQPAAAAPTTTTVLPTLEDPKLSLYAHVRLG

>Catharus_ustulatus

GGAEPRVSL**HDCVSCGSRC**RLRGGVPVVRLGGLETPKPLPKSVPAPRPDPKLSLYAHVRLG

>Anolis_carolinensis

MLKPLYIPV**HDCLACSAHRC**KIHKQLLVRLIRCPPSMLPVSHLRHSRRSRVQQDYSFMKKHNMTWLDRRSLVVNLGKRVPLDWVGLQTDKVSENGDHQLHSSNVELSQGIEEHPTGTVENHTEVTEPLASGEQAPFRSSDPLSSSQLLYRTRSMARRQAKLLPKSQLALPAVDPKLAQYAHVNLE

>Pogona_vitticeps

MLKPLYIPL**HDCLACGARQC**KIHKQLLVRLLQCPPSMLPASHLRRSLRSKARYSRAPQGCSVTEKHKSPSSSWTNQCNLVVNLGKRVPLNLWMNRKAVSGTGQREPYSRNGAFSQGPKERHEGDEEVTGPYESGEQAPLGSSGPLSYSQLLCRTRSMAKRQAQLLPKSQLFVPAVDPKLSQYAHVCLE

>Lacerta_agilis

ILKPLCIPL**HNCLGCTACSC**KLSKQPLVHLQRCPPSMFPASHLRRSSRKKSSHFETQQSFFITRTYNRPESPSWADQSDLVVNLGKRVPLHWEGLHRQAVRGIGEREPQSSNGELSQGAEEHHTGDVKNPAEIMGSHGSGSGELYSGDQEILGSTSSSSSGQVLCRTRSMTRRQTWVLPKPQVSLSAVDPKLSQYAHVRLE

>Podarcis_muralis

ILKPLCIPL**HNCLGCTARSC**KLSKQPLVHLQRCPPSMFPASHLRRSSRRKSSHFETQQSFFFTRTYNRPESPSWADQSDLVVNLGKRVPLHWEGLRREAVRGIGEREPHSSNGELSQGAEEHHTGDVKNPAEIMGSHGSGSGELYSGDQEILGSTGSSSSGQVLCRTRSMTRRQTWDLPKPQVSLLAVDPKLSQYAHVRLE

>Python_bivittatus

MLKPLYIPL**HNCLACRARRC**KLRKQPLVCLLRCPPPVLPVLHLRQSLRNKNCYSKAQQHCSLIKVHSCPGSLGLAGQCSLVVNLGKRIPLDWARFHREVVCDVKERKPPITNGEFSQGTEEYDSRDMESHADVIETRESGSGVLHSGQQVPLDSSGPLPSRQFLCRTRSMAQRQAQVLPRSQLSLATVDPKLSQYAHVRLE

>Alligator_sinensis

ALRHLRIPL**HNCVACRGIGRGC**RLRKELAVSLVRCLPGASAPETAVAQSSEALEHHPALYQHPPSSTEPSIPSSLGSLDLLRLKTPSLTRVFCVELGDTRVLASQDRGQGPGIGPPMLGVSSTPGEASPGAVPHSRDVVPPGGVVGHCPLAPQLLCRTRSMVKAVASCPAWPLEALAAAGRANPHPLPDPKLSLYAHVRLE

>Xenopus_laevis

VLRDVRIIL**HNCKRCNMASRSKSQHEGKCC**KLGKVPLVSILREDLSPEKLKFRLICPPTIQTANIECNAKDRSLPEAGSINMEPITTAEVCEHVSFSPLPSHNGDEDSFYEPEIPGSLLGPESPSSVLESGSEDGLSGPNNDEPISNNLNMECPPLEPIVKDTVYPYDGSKMSVSHLHALKQFGITRYIKVDLRKHVDLQSEKSQPDKSSSAANRKLNPNHIHSHQPLRSDDHLLANLNAKAQNKVVLRKDVALESEKPRPDKSSSATNRKPNLNHIHSQLPIRSDDHLPANFNTEAQNNTVVSPTAKTPISEALNGSPDRKVSSLRSRSVTFRETSDNKITLFKRRRSSVKPGVQCVKLNGHVKLTGQIVYSKLDPPTKAVAKHLTHTMHSDPKLLLKPYVQLG

>Xenopus_tropicalis

VLRDVRIIL**HNCKKCNMASHPKHQHDGQCC**KLGKEPLVSLRREDLSPEKLKFRLSCHGGSCSPTIQTANIECNAKDCPLPEAEKASAEQVTSAEVCEHLSFSPVPSLSGDDHSFYEPEIPASLLGPDSPSAEPLVKEPLSSVCPPPTVYPHNNNNGDIMSALHHQALKQFGITHYIKVGLSKDVRSLEKSGKNPADSSSLAAEPSDNCIHSQHPVTSDDRLAGNLTDETQGNPVSSPAKTQRCEALNGSLAPKLFSLRSRPVSFKASETSDNKITLFKRSSSSSAKPGVQYVKLNGHVKLTGQLVPSEPHAPNAARARPLADTMRSDPKLLLKPYVELG

>Hymenochirus_boettgeri

VLRDIRVIL**HNCKKCHATSHFQHLHERQCC**KLGKEPVVSLLREDLSPEKLKFRLHCSATYEASLTTPTVRFDRNAKDVCLTEVEVGESMVEQDTIAKVCESLSFSSVLSHSSDDDTFSEPEMPGSFLGPGSPSSDPPVQIQEAESNTIDTDFFPFESAAKDTVHPQNNADVSQFTLDSLNKQFGITHYVTVDLGKSIKRGVFNNSLAANVKPRDDHGLLPPAENTNGNLLASLDNETQSHTVNEPNKSKKNEALSESLSHKALSLRSRPVILKEQEASENSISLKQKRSCVNPNLKCLKINGHVKVTGQVVNSEPHQVKVAKTKNLNDPLNLDPKLLLKPYVELG

>Rana_temporaria

IIRDIRINL**HNPKCSRYSSAAGTPSEVHGC**KLSKEPVVRLSRQNVSPDKLSFYQAVDRALHNSSRTSLQGKTNALEKNAKDHTLNCALFNEPNVVEEHLDLEYLSATTSCCRDELDVDVYENQNCVSDSISNSDRLSTNGTDISMVQPEFDILASPSNSNRLCSAVVSQMQDDNFPPAASLPENTVNLNNSSFKLFSDSVSLKQLGLTHYVTVNLSKSRVSGAEYSNTPSFFHSDVRKHKRNRNQKPFTGFSHDQIVTKNANESPVIAENGSLTEPADINVKPRTGTCTNEVFSLQSTPTMFKVPNKRRKTTFHKKRRTTHLDFNGLIKVTGKHRISRPERRHSAPHALLSIEMQSDPKLSLKPYVELG

>Nanorana_parkeri

IIRDVRINL**HNSIKCSRYSSAAATPCEVHGC**KLGKEPVVRLRRQNASPDRLRFYQGSDNVLHKSSQTSLHGKTDTLEKDAKDHTLNCVLFNEHSVMEECLNPECPSASTSVCVDELDIEVIEHHENCASNFISNSDRLNANGAHTSMVQPALDVLVSPLDSNRLCSNVVSQMQEADNFPPARSLPESTVNLNENSSRLTFSDSMSPKQFGLTHYVTVNLSKFRVSGPEYSSTASAFHSDIGKHKRSYNQKPFTGITHEAVLATNAIESQVMDANSSLTEPVGINVKHSTRTYTNKVFSLQSRPIMSKGLNEKSASHKKRQTTHLDFNGHVKVTGKHQSSRLERHHSAPHKLLSMEMQSDPKLSLKPYVELS

**FASTA files for corresponding linker sequences (used in Figure 2b)**

>Homo_sapiens

GQPPHARWAPQQDWHWARRYG

>Mus_musculus

HLRPQTRWTPQQDWYWARRYG

>Canis_familiaris

GPAPRAHWAPQQDWHWARRYG

>Bubalus_bubalis

VQAPRARWAPQQDWHWARRYG

>Cavia_porcellus

GLPHSARWAPQQDWHWARHYG

>Coturnix_japonica

CRLQPPEPPPPQPTTTATPAVLPPREDPK

>Phasianus_colchicus

CRLPPPEPPAAAAAPRPTPPTPSLLFPLQDPK

>Gallus_gallus

CAAAPAGAPPPQPTAATPPAVLPPLEDPK

>Numida_meleagris

CRQPPAAPLPEPPPPQPAAAAPTTTTVLPTLEDPK

>Catharus_ustulatus

GGLETPKPLPKSVPAPRPDPK

>Anolis_carolinensis

IRCPPSMLPVSHLRHSRRSRVQQDYSFMKKHNMTWLDRRSLVVNLGKRVPLDWVGLQTDKVSENGDHQLHSSNVELSQGIEEHPTGTVENHTEVTEPLASGEQAPFRSSDPLSSSQLLYRTRSMARRQAKLLPKSQLALPAVDPK

>Pogona_vitticeps

LQCPPSMLPASHLRRSLRSKARYSRAPQGCSVTEKHKSPSSSWTNQCNLVVNLGKRVPLNLWMNRKAVSGTGQREPYSRNGAFSQGPKERHEGDEEVTGPYESGEQAPLGSSGPLSYSQLLCRTRSMAKRQAQLLPKSQLFVPAVDPK

>Lacerta_agilis

QRCPPSMFPASHLRRSSRKKSSHFETQQSFFITRTYNRPESPSWADQSDLVVNLGKRVPLHWEGLHRQAVRGIGEREPQSSNGELSQGAEEHHTGDVKNPAEIMGSHGSGSGELYSGDQEILGSTSSSSSGQVLCRTRSMTRRQTWVLPKPQVSLSAVDPK

>Podarcis_muralis

QRCPPSMFPASHLRRSSRRKSSHFETQQSFFFTRTYNRPESPSWADQSDLVVNLGKRVPLHWEGLRREAVRGIGEREPHSSNGELSQGAEEHHTGDVKNPAEIMGSHGSGSGELYSGDQEILGSTGSSSSGQVLCRTRSMTRRQTWDLPKPQVSLLAVDPK

>Python_bivittatus

MLRCPPPVLPVLHLRQSLRNKNCYSKAQQHCSLIKVHSCPGSLGLAGQCSLVVNLGKRIPLDWARFHREVVCDVKERKPPITNGEFSQGTEEYDSRDMESHADVIETRESGSGVLHSGQQVPLDSSGPLPSRQFLCRTRSMAQRQAQVLPRSQLSLATVDPK

>Alligator_sinensis

VRCLPGASAPETAVAQSSEALEHHPALYQHPPSSTEPSIPSSLGSLDLLRLKTPSLTRVFCVELGDTRVLASQDRGQGPGIGPPMLGVSSTPGEASPGAVPHSRDVVPPGGVVGHCPLAPQLLCRTRSMVKAVASCPAWPLEALAAAGRANPHPLPDPK

>Xenopus_laevis

REDLSPEKLKFRLICPPTIQTANIECNAKDRSLPEAGSINMEPITTAEVCEHVSFSPLPSHNGDEDSFYEPEIPGSLLGPESPSSVLESGSEDGLSGPNNDEPISNNLNMECPPLEPIVKDTVYPYDGSKMSVSHLHALKQFGITRYIKVDLRKHVDLQSEKSQPDKSSSAANRKLNPNHIHSHQPLRSDDHLLANLNAKAQNKVVLRKDVALESEKPRPDKSSSATNRKPNLNHIHSQLPIRSDDHLPANFNTEAQNNTVVSPTAKTPISEALNGSPDRKVSSLRSRSVTFRETSDNKITLFKRRRSSVKPGVQCVKLNGHVKLTGQIVYSKLDPPTKAVAKHLTHTMHSDPK >Xenopus_tropicalis

RREDLSPEKLKFRLSCHGGSCSPTIQTANIECNAKDCPLPEAEKASAEQVTSAEVCEHLSFSPVPSLSGDDHSFYEPEIPASLLGPDSPSAEPLVKEPLSSVCPPPTVYPHNNNNGDIMSALHHQALKQFGITHYIKVGLSKDVRSLEKSGKNPADSSSLAAEPSDNCIHSQHPVTSDDRLAGNLTDETQGNPVSSPAKTQRCEALNGSLAPKLFSLRSRPVSFKASETSDNKITLFKRSSSSSAKPGVQYVKLNGHVKLTGQLVPSEPHAPNAARARPLADTMRSDPK

>Hymenochirus_boettgeri

LREDLSPEKLKFRLHCSATYEASLTTPTVRFDRNAKDVCLTEVEVGESMVEQDTIAKVCESLSFSSVLSHSSDDDTFSEPEMPGSFLGPGSPSSDPPVQIQEAESNTIDTDFFPFESAAKDTVHPQNNADVSQFTLDSLNKQFGITHYVTVDLGKSIKRGVFNNSLAANVKPRDDHGLLPPAENTNGNLLASLDNETQSHTVNEPNKSKKNEALSESLSHKALSLRSRPVILKEQEASENSISLKQKRSCVNPNLKCLKINGHVKVTGQVVNSEPHQVKVAKTKNLNDPLNLDPK

>Rana_temporaria

SRQNVSPDKLSFYQAVDRALHNSSRTSLQGKTNALEKNAKDHTLNCALFNEPNVVEEHLDLEYLSATTSCCRDELDVDVYENQNCVSDSISNSDRLSTNGTDISMVQPEFDILASPSNSNRLCSAVVSQMQDDNFPPAASLPENTVNLNNSSFKLFSDSVSLKQLGLTHYVTVNLSKSRVSGAEYSNTPSFFHSDVRKHKRNRNQKPFTGFSHDQIVTKNANESPVIAENGSLTEPADINVKPRTGTCTNEVFSLQSTPTMFKVPNKRRKTTFHKKRRTTHLDFNGLIKVTGKHRISRPERRHSAPHALLSIEMQSDPK

>Nanorana_parkeri

RRQNASPDRLRFYQGSDNVLHKSSQTSLHGKTDTLEKDAKDHTLNCVLFNEHSVMEECLNPECPSASTSVCVDELDIEVIEHHENCASNFISNSDRLNANGAHTSMVQPALDVLVSPLDSNRLCSNVVSQMQEADNFPPARSLPESTVNLNENSSRLTFSDSMSPKQFGLTHYVTVNLSKFRVSGPEYSSTASAFHSDIGKHKRSYNQKPFTGITHEAVLATNAIESQVMDANSSLTEPVGINVKHSTRTYTNKVFSLQSRPIMSKGLNEKSASHKKRQTTHLDFNGHVKVTGKHQSSRLERHHSAPHKLLSMEMQSDPK
