# Supplementary figures and images for: KMT5C leverages disorder to optimize cooperation with HP1 for heterochromatin retention
Source: EMBO Rep. 2024 Nov 19;26(1):153–74. doi: 10.1038/s44319-024-00320-5 (PMC11723951; doi:10.1038/s44319-024-00320-5)

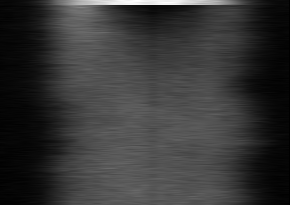

Supplement: Supplementary file 23 — Source data Fig. 1 [file 44319_2024_320_MOESM23_ESM.zip › Figure 1/A/KMT5C_partial_Kymograph.tif]

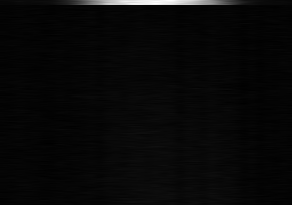

Supplement: Supplementary file 23 — Source data Fig. 1 [file 44319_2024_320_MOESM23_ESM.zip › Figure 1/A/KMT5C_total_Kymograph.tif]

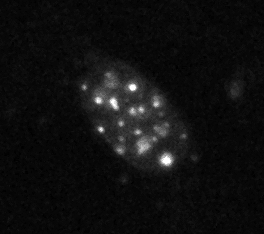

Supplement: Supplementary file 23 — Source data Fig. 1 [file 44319_2024_320_MOESM23_ESM.zip › Figure 1/B/Microscope Image (KMT5C iFRAP).tif]

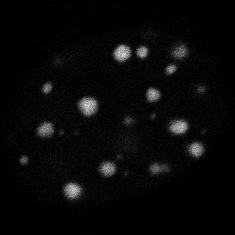

Supplement: Supplementary file 26 — Source data Fig. 5 [file 44319_2024_320_MOESM26_ESM.zip › Figure 5/D/Microscope Image Files/D5 CBX5MBD.tif]

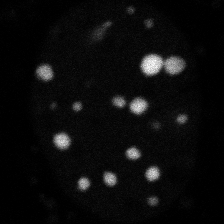

Supplement: Supplementary file 26 — Source data Fig. 5 [file 44319_2024_320_MOESM26_ESM.zip › Figure 5/D/Microscope Image Files/W8 CBX5MBD.tif]

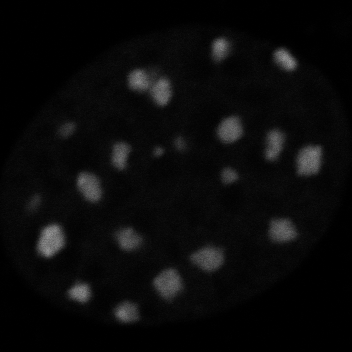

Supplement: Supplementary file 26 — Source data Fig. 5 [file 44319_2024_320_MOESM26_ESM.zip › Figure 5/E/Microscope Image Files/D5 CBX5MBD-co.tif]

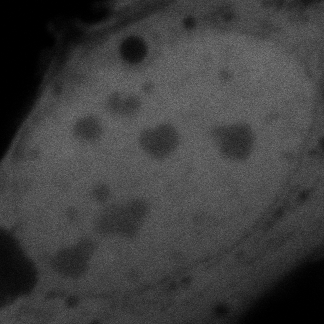

Supplement: Supplementary file 26 — Source data Fig. 5 [file 44319_2024_320_MOESM26_ESM.zip › Figure 5/E/Microscope Image Files/D5 im3.tif]

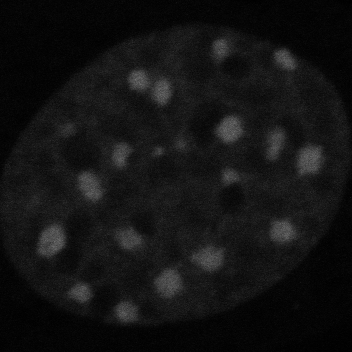

Supplement: Supplementary file 26 — Source data Fig. 5 [file 44319_2024_320_MOESM26_ESM.zip › Figure 5/E/Microscope Image Files/D5 im3-co.tif]

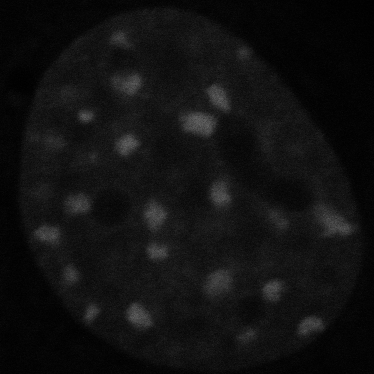

Supplement: Supplementary file 26 — Source data Fig. 5 [file 44319_2024_320_MOESM26_ESM.zip › Figure 5/E/Microscope Image Files/W8 im3.tif]

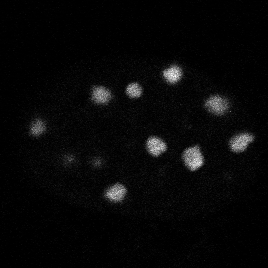

Supplement: Supplementary file 26 — Source data Fig. 5 [file 44319_2024_320_MOESM26_ESM.zip › Figure 5/F/Microscope Image Files/D5 CBX5MBD-co.tif]

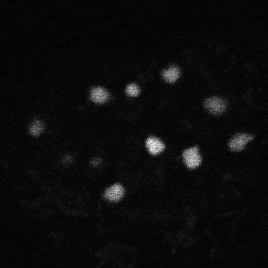

Supplement: Supplementary file 26 — Source data Fig. 5 [file 44319_2024_320_MOESM26_ESM.zip › Figure 5/F/Microscope Image Files/D5 HRD-co.tif]

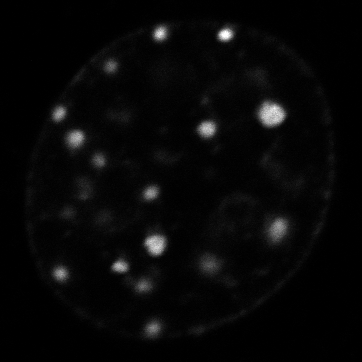

Supplement: Supplementary file 26 — Source data Fig. 5 [file 44319_2024_320_MOESM26_ESM.zip › Figure 5/F/Microscope Image Files/W8 HRD.tif]

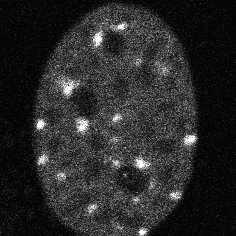

Supplement: Supplementary file 26 — Source data Fig. 5 [file 44319_2024_320_MOESM26_ESM.zip › Figure 5/G/Microscope Image Files/D5 CBX5MBD-co.tif]

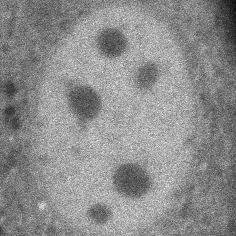

Supplement: Supplementary file 26 — Source data Fig. 5 [file 44319_2024_320_MOESM26_ESM.zip › Figure 5/G/Microscope Image Files/D5 delta23-co.tif]

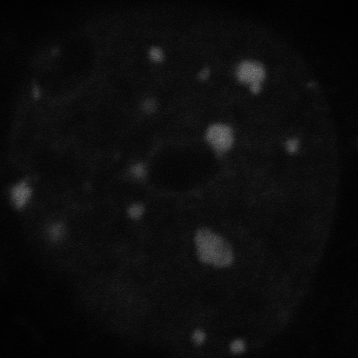

Supplement: Supplementary file 26 — Source data Fig. 5 [file 44319_2024_320_MOESM26_ESM.zip › Figure 5/H/Microscope Image Files/W8 HRD-co.tif]

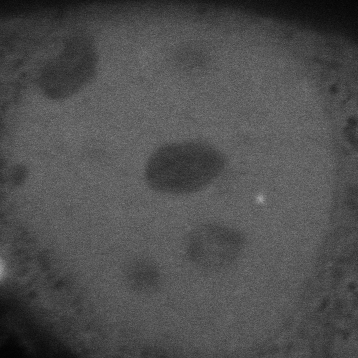

Supplement: Supplementary file 26 — Source data Fig. 5 [file 44319_2024_320_MOESM26_ESM.zip › Figure 5/H/Microscope Image Files/W8 im3-co.tif]

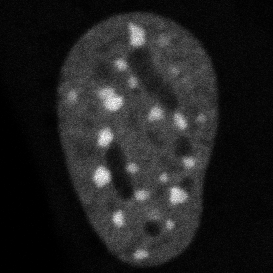

Supplement: Supplementary file 27 — Source data Fig. 6 [file 44319_2024_320_MOESM27_ESM.zip › Figure 6/C/Microscope Image Files/H3K9me-/HRD-IDR1.tif]

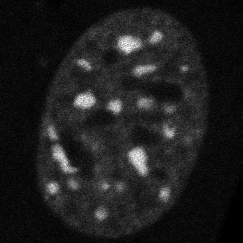

Supplement: Supplementary file 27 — Source data Fig. 6 [file 44319_2024_320_MOESM27_ESM.zip › Figure 6/C/Microscope Image Files/H3K9me-/HRD-IDR2.tif]

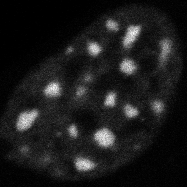

Supplement: Supplementary file 27 — Source data Fig. 6 [file 44319_2024_320_MOESM27_ESM.zip › Figure 6/C/Microscope Image Files/H3K9me-/HRD-IDR3.tif]

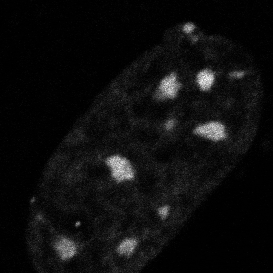

Supplement: Supplementary file 27 — Source data Fig. 6 [file 44319_2024_320_MOESM27_ESM.zip › Figure 6/C/Microscope Image Files/H3K9me-/HRD-IDR4.tif]

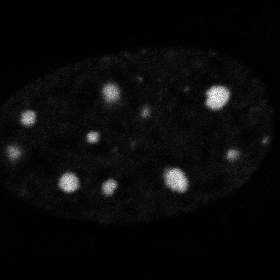

Supplement: Supplementary file 27 — Source data Fig. 6 [file 44319_2024_320_MOESM27_ESM.zip › Figure 6/C/Microscope Image Files/H3K9me-/HRD-IDR5.tif]

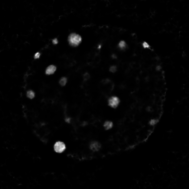

Supplement: Supplementary file 27 — Source data Fig. 6 [file 44319_2024_320_MOESM27_ESM.zip › Figure 6/C/Microscope Image Files/H3K9me3+/HRD-IDP1.tif]

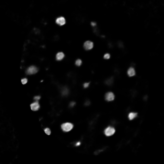

Supplement: Supplementary file 27 — Source data Fig. 6 [file 44319_2024_320_MOESM27_ESM.zip › Figure 6/C/Microscope Image Files/H3K9me3+/HRD-IDP2.tif]

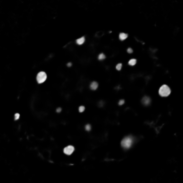

Supplement: Supplementary file 27 — Source data Fig. 6 [file 44319_2024_320_MOESM27_ESM.zip › Figure 6/C/Microscope Image Files/H3K9me3+/HRD-IDP3.tif]

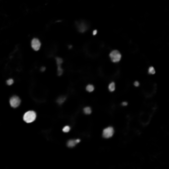

Supplement: Supplementary file 27 — Source data Fig. 6 [file 44319_2024_320_MOESM27_ESM.zip › Figure 6/C/Microscope Image Files/H3K9me3+/HRD-IDP4.tif]

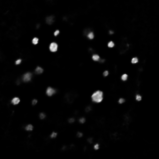

Supplement: Supplementary file 27 — Source data Fig. 6 [file 44319_2024_320_MOESM27_ESM.zip › Figure 6/C/Microscope Image Files/H3K9me3+/HRD-IDP5.tif]
