# Supplementary material for: KMT5C leverages disorder to optimize cooperation with HP1 for heterochromatin retention
Source: EMBO Rep. 2024 Nov 19;26(1):153–74. doi: 10.1038/s44319-024-00320-5 (PMC11723951; doi:10.1038/s44319-024-00320-5)
Supplement: Supplementary file 28 — Expanded View Figures [file 44319_2024_320_MOESM28_ESM.pdf]

Expanded View Figures

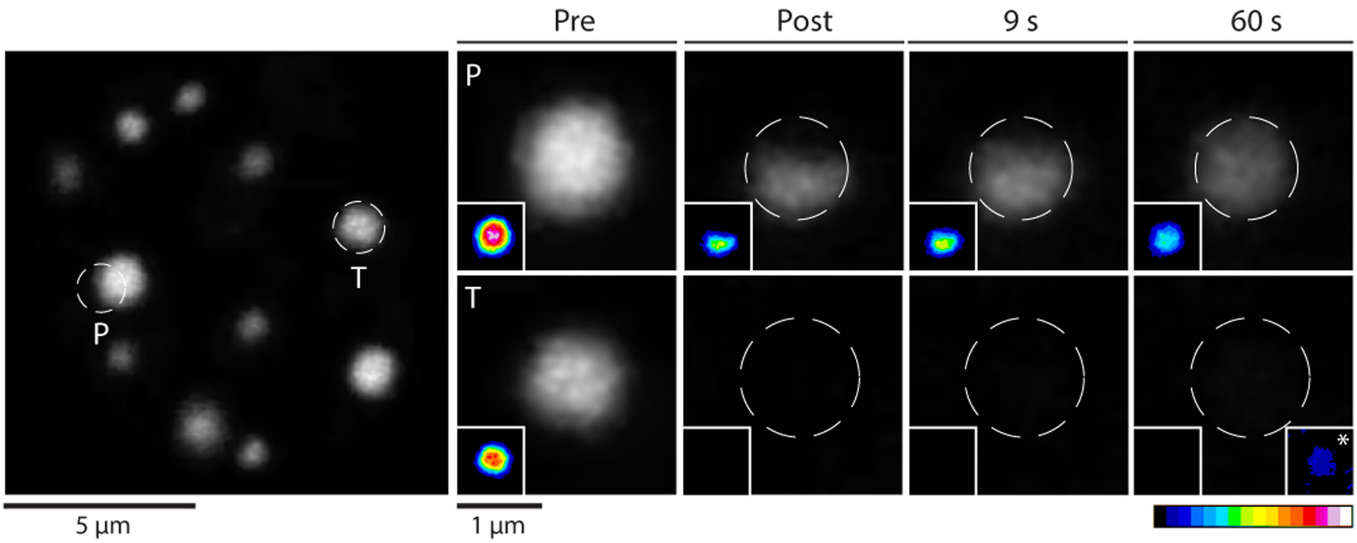

Figure EV1. Characterization of HRD dynamics within constitutive heterochromatin compartments.

(**A**) Time-lapse series of total (T) and partial (P) HRD-mEmerald fluorescence recovery after photobleaching (Movie EV2) in mouse NMuMG immortalized breast epithelial cells ($n = 45$) (see Methods). Representative images are shown for pre-bleach, post-bleach (0.3 s), 9 s, and 60, and represent transiently transfected cells. Insets show fluorescence intensity using 16-color LUT. Asterisk denotes Hoechst channel to indicate presence of chromocenter.

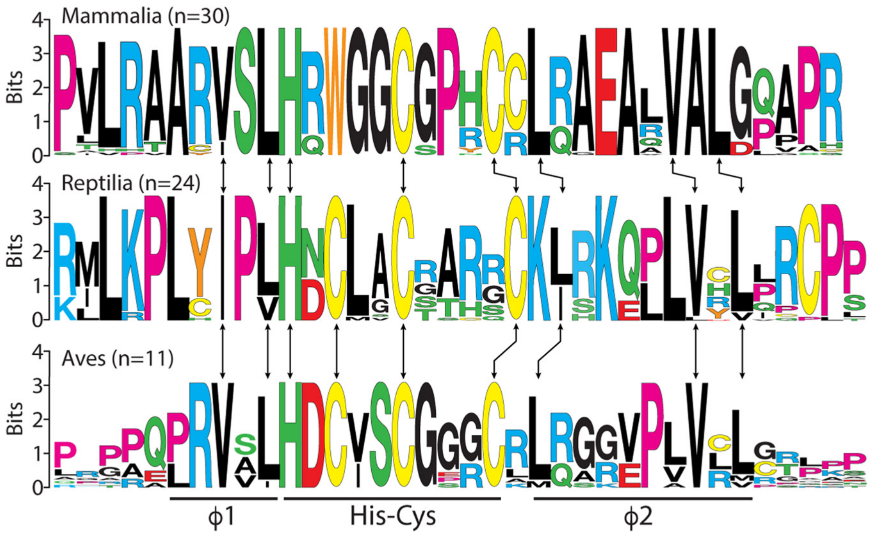

Figure EV2. WebLogo depiction for the HRD CR1 from representative mammals, birds, and reptiles.

The WebLogo (Crooks et al, 2004) is derived from the indicated number of sequences in each species. Key conserved features between classes are noted with arrows to account for differences in spacing. The frog version was omitted because the corresponding sequences show more variability in spacing between the histidine and cysteine residues. Detailed information regarding CR2 is provided in Fig. 5.

© The Author(s)

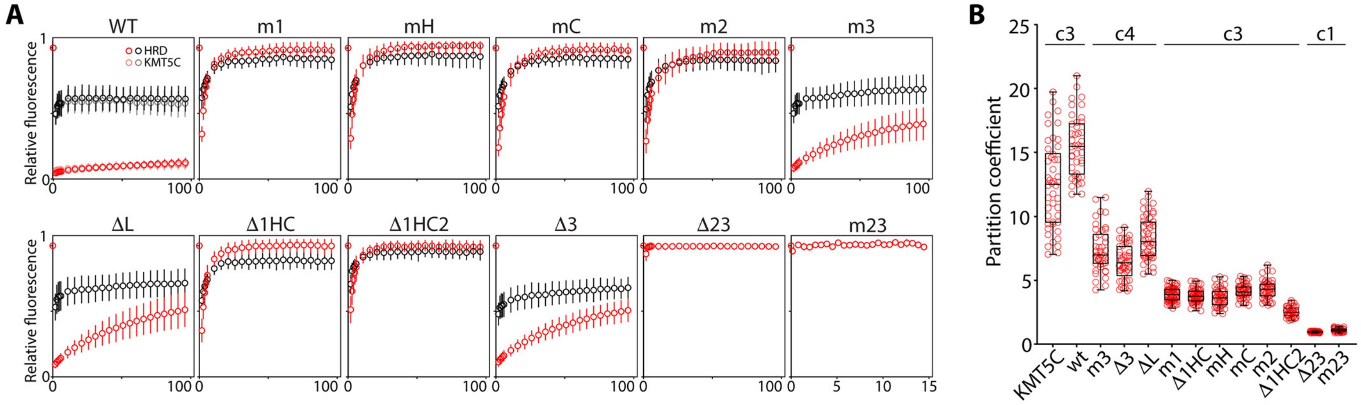

Figure EV3. Complete FRAP and partitioning data for KMT5C, HRD, and corresponding derivatives.

(**A**) FRAP curves (relative fluorescence vs. time) depict profiles for total (red) and partial (black) photobleaching of chromocenters for KMT5C and the HRD (superimposed), m1 (Φ1 mutant), mH (histidine mutant), mC (cysteine mutant), m2 (Φ2 mutant), m3 (Φ3 mutant), ΔL (linker deletion), Δ1HC (Φ1-histidine-cysteine deletion), Δ1HC2 (Φ1-histidine-cysteine-Φ2 deletion), Δ3 (Φ3 deletion), Δ23 (Φ2-Φ3 deletion), m23 (Φ2-Φ3 mutation). With the exception of m23, where recovery was monitored with higher sampling over a 15-second period, the remaining graphs show recovery from 0 to 100 s. (**B**) Partition coefficients (normalized chromocenter intensity divided by normalized nucleoplasmic intensity; see methods) are shown for the indicated proteins. Clusters (c1–c4) correspond to those in Fig. 3c. In FRAP plots, vertical lines correspond to the standard deviation of the mean. For box plots, vertical lines indicate the bounds of the box and whiskers (minimum to maximum) and the box corresponds to middle 50% of PC values (Q1–Q3) with the median indicated by a horizontal line. For FRAP and PC data, three separate experiments were conducted with a minimum of 15 cells each.

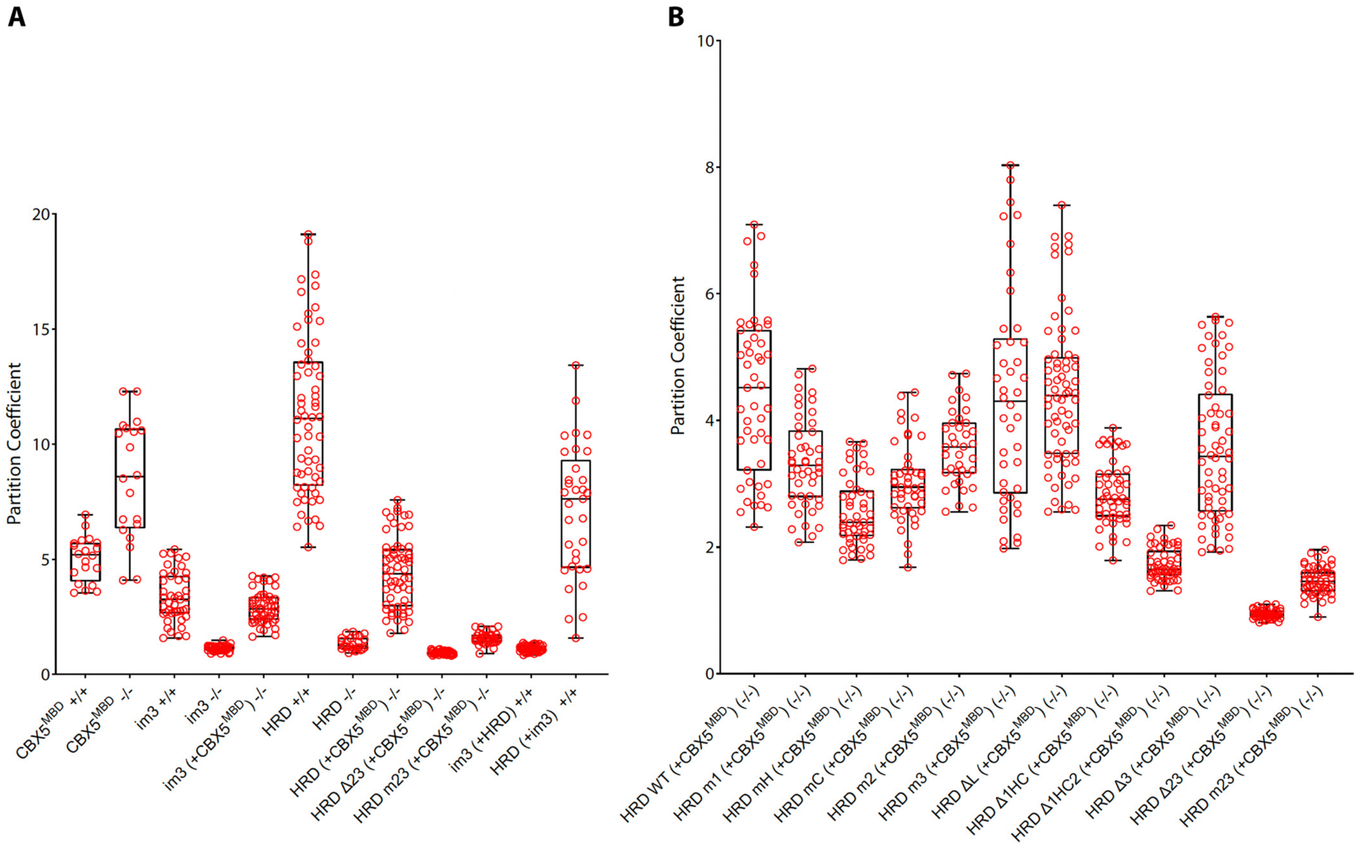

Figure EV4. Partition coefficient data for *Suv39h1/2* (wild-type and null) and CBX5^{MBD} rescue experiments.

(**A**) Partition coefficients (normalized chromocenter intensity divided by normalized nucleoplasmic intensity; see methods) are shown for the indicated proteins (single or co-transfection) and cell conditions (*Suv39h1/2* wild-type and null MEFs) from Fig. 5. (**B**) Partition coefficient data for CBX5^{MBD} rescue of all remaining mutant proteins from Fig. 3. For box plots, vertical lines indicate bounds of box and whiskers (minimum to maximum) and the box corresponds to middle 50% of PC values (Q1–Q3) with the median indicated by a horizontal line. For PC data, three separate experiments were conducted with a minimum of 15 cells each.

 © The Author(s)

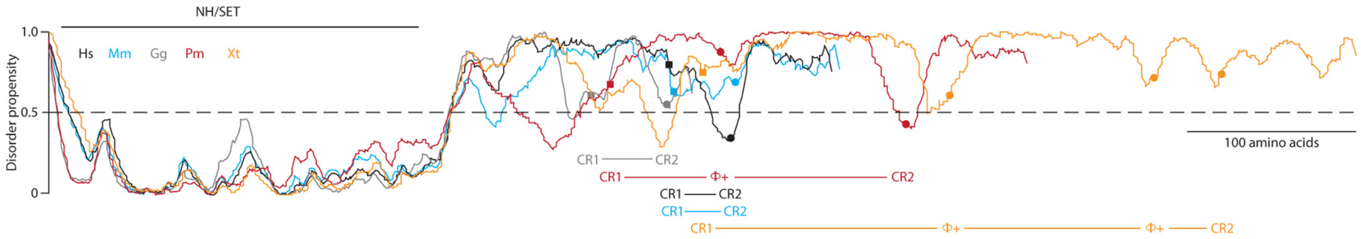

Figure EV5. Disorder plot for representative KMT5C orthologs.

Graph depicts Metapredict disorder propensity (0 being lowest and 1 being highest) for KMT5C orthologs from *Homo sapiens* (Hs), *Mus musculus* (Mm), *Gallus gallus* (Gg), *Podarcis muralis* (Pm), and *Xenopus tropicalis* (Xt) that have been anchored to the amino-terminal catalytic region (NH/SET). For each species, the location of the CR1 region is noted by a *square* and the CR2 region by a *circle*. For the longer Pm and Xt proteins, the presence of potential additional hydrophobic motifs (Φ+) in the region between the CR1 and CR2 motifs are noted.

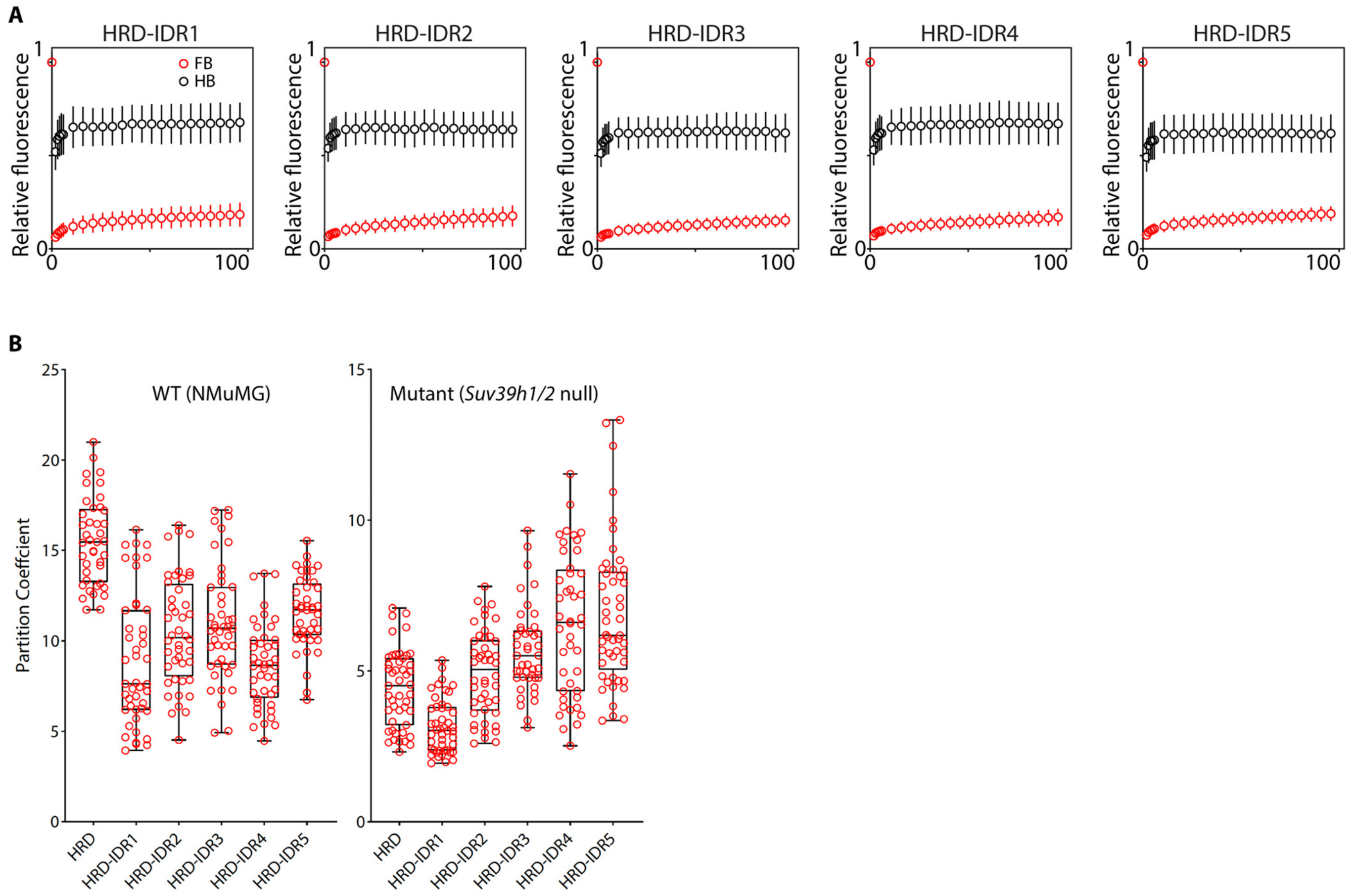

Figure EV6. Complete FRAP and partitioning data for HRD chimeras.

(**A**) FRAP curves (relative fluorescence vs. time) depict profiles for total (red) and partial (black) photobleaching for the HRD-IDR chimeras in NMuMG cells from 0 to 100 s. (**B**) Partition coefficients (normalized chromocenter intensity divided by normalized nucleoplasmic intensity; see methods) are shown for the indicated proteins in both wild-type and *Suv39h1/2* null conditions with co-expression of CBX5^{MBD}. In FRAP plots, vertical lines correspond to the standard deviation of the mean. For box plots, vertical lines indicate the bounds of the box and whiskers (minimum to maximum) and the box corresponds to middle 50% of PC values (Q1–Q3) with the median indicated by a horizontal line. For FRAP and PC data, three separate experiments were conducted with a minimum of 15 cells each.

© The Author(s)
